# Properties of Cemented Filling Materials Prepared from Phosphogypsum-Steel Slag–Blast-Furnace Slag and Its Environmental Effect

**DOI:** 10.3390/ma17143618

**Published:** 2024-07-22

**Authors:** Kai Li, Lishun Zhu, Zhonghu Wu, Xiaomin Wang

**Affiliations:** 1 Guiyang Water Environment Group Co., Ltd., Guiyang 550025, China; likai18985199981@163.com; 2 College of Civil Engineering, Guizhou University, Guiyang 550025, China; zhulishun0627@163.com (L.Z.); wuzhonghugzu@163.com (Z.W.)

**Keywords:** all-solid waste backfill materials, phosphogypsum, toxic leaching, micromechanisms, steel slag, ground granulated blast-furnace slag

## Abstract

Phosphogypsum (PG) occupies a large amount of land due to its large annual production and low utilization rate, and at the same time causes serious environmental problems due to toxic impurities. PG is used for mine backfill, and industrial solid waste is a curing agent for PG, which can save the filling cost and reduce environmental pollution. In this paper, PG was used as a raw material, combined with steel slag (SS) and ground granulated blast-furnace slag (GGBS) under the action of an alkali-activated agent (NaOH) to prepare all-solid waste phosphogypsum-based backfill material (PBM). The effect of the GGBS to SS ratio on the compressive strength and toxic leaching of PBM was investigated. The chemical composition of the raw materials was obtained by XRF analysis, and the mineral composition and morphology of PBM and its stabilization/curing mechanism against heavy metals were analyzed using XRD and SEM-EDS. The results showed that the best performance of PBM was achieved when the contents of PG, GGBS, and SS were 80%, 13%, and 7%, the liquid-to-solid ratio was 0.4, and the mass concentration of NaOH was 4%, with a strength of 2.8 MPa at 28 days. The leaching concentration of fluorine at 7 days met the standard of groundwater class IV (2 mg/L), and the leaching concentration of phosphorus was detected to be less than 0.001 mg/L, and the leaching concentration of heavy metals met the environmental standard at 14 d. The hydration concentration in PBM met the environmental standard. The hydration products in PBM are mainly ettringite and C-(A)-S-H gel, which can effectively stabilize the heavy metals in PG through chemical precipitation, physical adsorption, and encapsulation.

## 1. Introduction

Phosphogypsum (PG) is a by-product from the production of phosphoric acid, usually in the form of calcium sulfate dihydrate [1], and small amounts of impurities, such as organic matter, fluoride, and acidic impurities [2,3]. Each production of 1 ton of phosphoric acid produces about 5 tons of PG; the global phosphate fertilizer industry PG annual production is about 200–300 million tons, of which about 25% originates in China [4,5,6].

**Figure 1:**
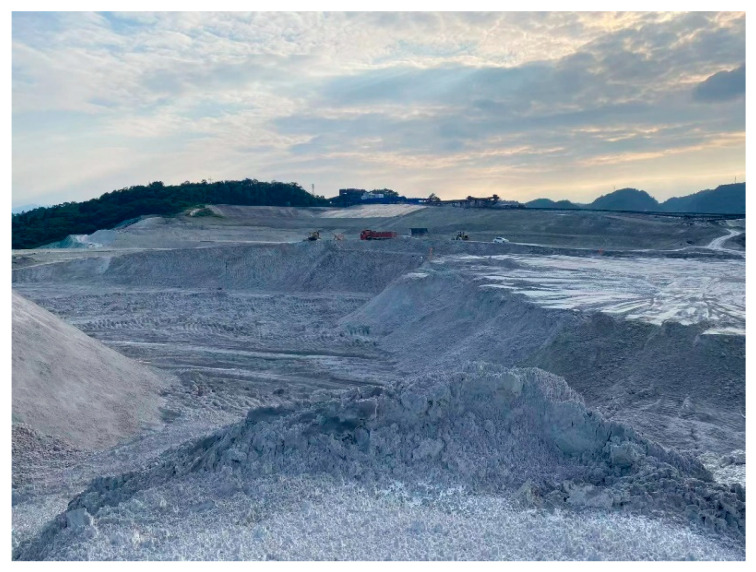
Large stockpiles of PG.

To face the problem of a large amount of PG produced globally every year, many ways of utilizing PG have been studied in China and abroad. For example, the use of PG as a setting retarder in silicate cement [7], the use of PG for fertilizer and soil improvement [8], for the production of building materials or road base materials [9,10], preparation of hollow unit masonry for walls and partitions [11], pre-treated for cementitious materials [12], non-fired bricks [13], etc. However, globally, only about 15% of phosphogypsum is utilized in these ways, and the consumption of phosphogypsum is very limited for the purpose of large-scale consumption and utilization of phosphogypsum. For some of these utilization routes, there is also some pretreatment of PG, such as heat treatment or washing [14,15], which may consume large amounts of energy or water resources, as well as produce secondary pollution and lead to increased costs. About 85% of PG is landfilled or discarded without any treatment on a large scale (Figure 1) [16]. This takes up a lot of land resources, and the stockpiled phosphogypsum contains many heavy metals and toxic impurities, which will penetrate into the soil with running water, seriously polluting the groundwater resources and the soil [17,18]. In addition, in other countries, phosphogypsum has problems with radioactive substances [19]. Fortunately, however, the production of phosphogypsum in China does not have this problem [20,21,22,23,24]. Therefore, it is necessary to explore economical and environmentally friendly ways to consume PG on a large scale and at a low cost.

In the process of mineral resources development, a large number of empty areas are left after ore mining. As an environmentally friendly mining method, fill mining has been widely used in underground mines [25,26]. In the backfill mining method, factors such as raw material cost, production process cost, and energy consumption of backfilling materials should be considered. In the mining area, a large amount of PG mixed with a curing agent is used to prepare a backfill material to support the surrounding area to create a safe underground environment and mitigate surface settlement [25]. The use of phosphogypsum for mine filling can not only solve the problem of large-scale surface storage but also reduce the cost of treating goaf and achieve resource recycling; reduce environmental pollution and landscape damage in mining areas; and reduce the risk of geological hazards during the mining process. PG contains many heavy metals and toxic impurities; for the reuse of PG, another key point is the curing effect of toxic substances in PG. PG contains phosphorus, fluorine, sulfur, and heavy metals, and these toxic substances may dissolve or leach out, and the eroded toxic substances are deposited in nearby soil or transferred to groundwater, and this contamination may be hazardous to human health [27,28]. Li et al. [22] investigated that impurities in PG can be well stabilized/consolidated in backfill materials. The curing agent is mixed with PG to form the backfill material. Throughout this transformative process, an array of physical and chemical reactions take place, orchestrating a symphony of ion exchange, hydration reactions, volcanic ash interactions, and the pivotal processes of flocculation and agglomeration. These intricate reactions give rise to the emergence of hydrated calcium silicate gels, hydrated calcium alumino-silicate gels, and ettringite, among other compounds, which permeate the pores of the PG particles. This treatment serves to fortify the mechanical integrity of the composite material while significantly diminishing its potential for leaching toxicity.

However, the cementing filler technology used in the vast majority of mines uses a curing agent that is basically cement, which is highly dependent on cement and consumes a large amount of energy in its production. Industrial solid waste as a raw material, instead of cement as a curing agent, can not only realize the resource utilization of industrial solid waste but can also reduce the production cost and energy consumption of the curing agent. GGBS and SS are widely used in cementitious materials [29,30]. Ground granulated furnace slag (GGBS) is a by-product in the process of ironmaking in the blast furnace, and its chemical composition mainly consists of CaO, SiO_2_, and Al_2_O_3_, and its chemical composition is basically the same as that of cement. GGBS itself is weakly water-hard and highly active, and it is a better material for preparing alkali-blasted materials; GGBS can even react with gypsum without the use of an activator [31,32]. However, fluoride and residual acids in PG prolong the setting time and weaken the mechanical strength during backfilling [20]. Therefore, in order to obtain sufficient reactivity, it is better to use an alkaline environment. Zheng et al. [24] used lime to activate GGBS and thus stabilize phosphogypsum, which stabilized PG to reach a compressive strength of 5–6 MPa at 14 days, and effectively stabilized fluorine and phosphorus in PG. Min et al. [33] stabilized PG using GGBS and composite silicate cement. A 120 d compressive strength of 4.08 MPa was achieved by curing at 9% slag doping, 6% cement, and 85% PG. Steel slag (SS) a by-product of the steelmaking process contains C_3_S, C_2_S, and CaO, etc., and belongs to the clinker making the slag reactive [34]. It has been shown that CaO can effectively fix P and free F in PG to form an alkaline system, and the alkaline system has the potential to fix heavy metal ions [23]. And SS can provide a certain amount of CaO to the PG backfill system, which helps to solidify the impurities. In addition, minerals such as calcium sulfate in PG and CaO and MgO in SS compensate for the volumetric shrinkage produced by alkali-activated slag [35,36].

Current research in PG is directed towards providing a new method to economically and environmentally friendly utilize large amounts of PG. In this study, phosphogypsum-based mine backfill material (PBM) was prepared by using PG as the main raw material and steel slag (SS) and ground granulated blast-furnace slag (GGBS) as the cementing components. The compressive strength of the PBM was evaluated, and the environmental behavior of impurities such as phosphorus, fluorine, and various metals from the PG was also monitored. The mechanism of strength growth of backfill materials and the curing mechanism of toxic impurities in PG were revealed by X-ray diffraction (XRD) and scanning electron microscope (SEM).

## 2. Materials and Methods

### 2.1. Raw Materials

**Figure 2:**
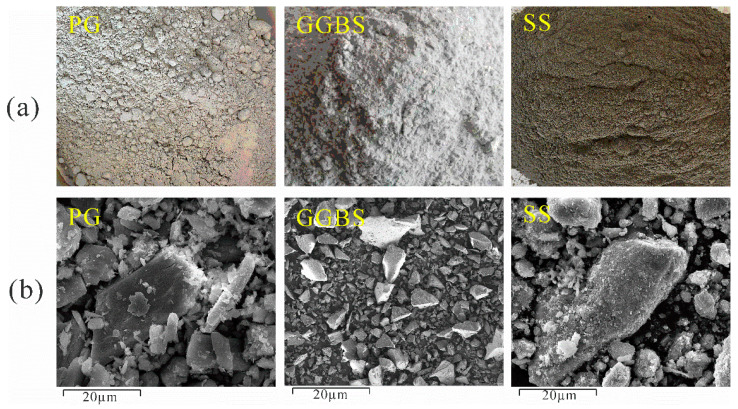
Raw materials: (**a**) Actual picture of raw materials; (**b**) SEM of raw materials.

The materials utilized in the experiments outlined in this paper encompass phosphogypsum (PG), steel slag (SS), and ground granulated blast-furnace slag (GGBS), respectively. The alkali-activated agent employed was solid sodium hydroxide (NaOH), procured from Inner Mongolia Yihua Chemical Co. Figure 2 shows the raw material and the SEM image of the raw material. PG was provided by a company in Guiyang, Guizhou, China. The PG subjected to experimentation was meticulously sieved, with a mesh size of 5 mm in diameter. Characterized by its gray-white powder form, the initial PG exhibited a moisture content of 17.6% and a density of 0.86. Upon scrutiny via scanning electron microscopy (SEM), the PG revealed a flake-like morphology with discernible prismatic angles. SS was produced in Gongyi City, Henan Province, with the appearance of black particles, moisture content of 1.12%, and density of 1.64. It was observed under SEM that the shape of SS particles was irregular, mainly lumpy and granular. GGBS was produced from a processing plant in Hebei Province, white powder, with a moisture content of 0.8% and a density of 1.04 Under SEM, it was observed that the shape of GGBS particles showed irregularity and angularity.

**Figure 3:**
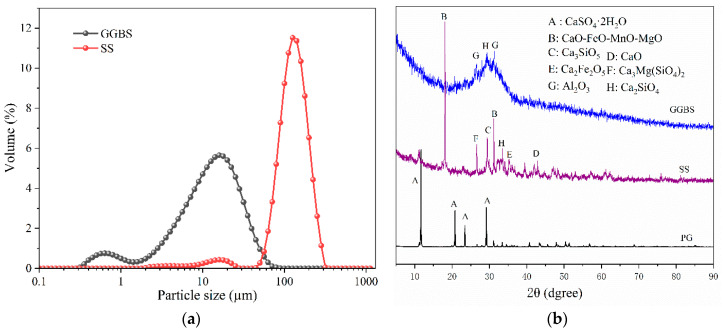
Particle size distribution and XRD analysis of raw materials: (**a**) Raw material particle size distribution; (**b**) XRD analysis of raw materials.

The chemical compositions of PG, GGBS, and SS were determined through X-ray fluorescence spectroscopy (XRF) analysis, as presented in Table 1. Mineral compositions were assessed using X-ray Diffractometry (XRD), with results depicted in Figure 3b. Analysis revealed that PG primarily comprises SO_3_ and CaO, accounting for over 96% of its composition, with the principal mineral phase identified as CaSO_4_·2H_2_O. SS is characterized by significant levels of CaO, MgO, SiO_2_, Al_2_O_3_, and Fe_2_O_3_, constituting more than 92% of its composition, crucial for initiating volcanic ash reactions in PBM materials. XRD patterns unveiled crystalline compounds within SS including CaO, CaSi_2_O_5_, and CaFe_2_O_4_. GGBS exhibits dominant SiO_2_ and CaO contents, comprising 34.50% and 34%, respectively, with CaO also serving as an essential excitation component within the system. Principal crystalline compounds in GGBS encompass Ca_2_SiO_4_ and Al_2_O_3_.

The results of the toxicity leaching test of in situ PG are shown in Table 2. The leaching concentration of the main heavy metal ions in PG was obtained by conducting three heavy metal leaching tests on PG and taking the average of the three test results, with reference to the Chinese groundwater quality class III standard (GB/T 14848-2017) [37], in which the leaching concentration of the element Mn exceeded the standard by 3 times, the leaching concentration of the element Ni was 58 times higher than the standard, the leaching concentration of the element As was 109 times higher than the standard, and the leaching concentration of the element Pb was also higher than the groundwater class III standard. Heavy metal elements also exceeded the groundwater class III standard, indicating that there is a risk of groundwater contamination from direct storage of PG without treatment. According to the “Standard for Groundwater Quality” (GB/T 14848-2017), the leaching concentration of heavy metals is within the range of Class III standard for groundwater, and the water body is not hazardous to the environment and human health.

### 2.2. Mix Proportions and Preparation of PBM

#### 2.2.1. Mix Proportions

The PBM consisted of PG as the main raw material. The molar concentration of NaOH alkali-activated agent was set to 1 mol/L [38]. SS and GGBS as the gelling component. The mass of the gelling component: PG was 2:8 [22]. Five groups of material ratios were set up for the test, with GGBS varying from 16% to 4% and SS from 4% to 16%. According to the Chinese standard (GB/T 39489-2020) [39], the slump requirement should be within the range of 180 mm~260 mm. In this study, the liquid–solid ratio was fixed at 0.4, and the slump was approximately 245 mm. The specific experimental program is shown in Table 3.

#### 2.2.2. Preparation of PBM

**Figure 4:**
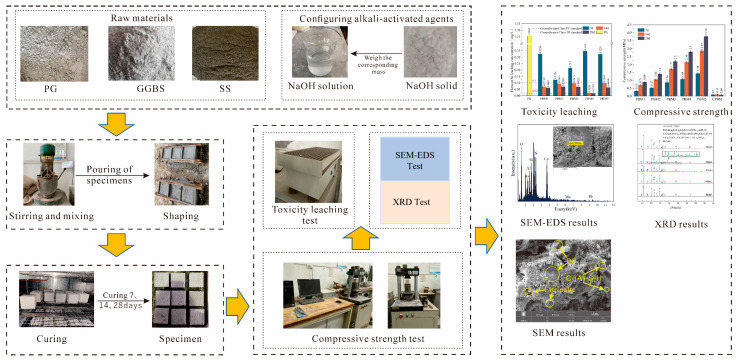
Production of specimens and testing procedures flow chart.

Backfill body specimen preparation process in accordance with JGJ/T 70-2009 [40] standard implementation, according to the table of the experimental program material ratio. First of all, weighed NaOH dissolved in distilled water to configure the Alkali-activated agent, then weighed the raw material, poured the material into the mixer dry stirring for 30 s, added the corresponding amount of water, and stirred for 60 s. Secondly, after mixing was completed, the slurry was poured into a feeler with a size of 70.7 × 70.7 × 70.7 mm for molding, and then the molded test specimen was put into a standard maintenance box for maintenance under standard maintenance conditions (temperature of 20 ± 3 °C, humidity of 90%) for 24 h and then demolded to obtain the test specimen, and then continue to place it in the curing box for curing. According to the Chinese standard JGJ/T 70-2009, unconfined compressive strength tests, toxicity leaching tests, and microstructure tests are conducted after curing for 7, 14, and 28 days. The specific process is shown in Figure 4.

### 2.3. Test Methods

#### 2.3.1. Compressive Strength Test

The PBM unconfined compressive strength test was conducted using a TYE-300D type cementitious sand compressive testing machine. Compressive strength tests were performed on PBM specimens with curing times of 7, 14, and 28 days. The loading rate of the instrument was fixed at 0.1 mm/min during the test, and the test was repeated three times for each group of tests, and the strength values used in the study were the average values calculated from the measured data. The unconfined compressive strength was used for microstructural analysis at the end of the test.

#### 2.3.2. Toxicity Leaching Test

The toxicity leaching test of PBM specimens was conducted in accordance with the Chinese standard “Solid Waste–Extraction procedure for leaching toxicity–Horizontal vibration method HJ-557-2010” [41]. Small fragments sieved through a 3 mm sieve were selected as experimental samples. The samples were mixed with distilled water at a ratio of 1:10 (g/mL) in 2 L extraction bottles. The bottle was placed on a horizontal shaker with an oscillation frequency of 110 ± 10 times/min and an amplitude of 40 mm for 8 h. Subsequently, it was left to stand horizontally for 16 h. The liquid was withdrawn and passed through a 0.45 µm membrane to obtain the filter solution. This solution was analyzed for heavy metal ions using an ICP-MS instrument.

#### 2.3.3. SEM Test

Analysis of micro-morphological differences in specimens with different ratios using SEM inspection. First, freshly crushed samples from the compressive test are dried in an oven at 40 °C, which must be below 70 °C to prevent decomposition of hydration products [21]. The dried samples are then cut into fresh sections with an area of about 0.5–1 cm^2^. Next, these cut nuggets are affixed to a metal carrier base and placed in the vacuum chamber of the gold sprayer for about 30 min to ensure that the vacuum requirements needed for testing are met. The surface of the samples was subsequently sprayed with gold to enhance its electrical conductivity. Finally, experiments were conducted using a COXEMEM-30PLUS-type scanning electron microscope with a set accelerating voltage of 0.3–30 kV.

#### 2.3.4. XRD Test

XRD is a technique used to identify the mineralogical components of materials and is widely used in many studies. Firstly, the crushed specimens after compressive strength testing were immersed in acetone solution to stop their hydration reaction and then dried in a vacuum oven at 50 °C to a constant weight. Subsequently, the dried specimens were ground into powder, sieved through a 74 μm sieve, and the powder was filled and pressed into the grooves of the test slides for XRD testing. The XRD tests were carried out using a Bruker D8 X-ray diffractometer employing Cu-Kα radiation with an operating current of 80 mA and an operating voltage of 60 kV. The scanning range of the test was set at 5–90° 2θ, and the scanning speed of the instrument was set at 5°/min.

## 3. Results and Discussion

### 3.1. Unconfined Compressive Strength

**Figure 5:**
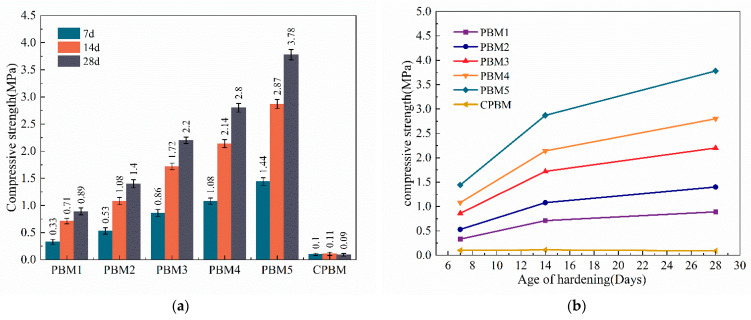
Effect of mixture ratios on the compressive strength of PBM: (**a**) Effect of ratio on strength; (**b**) Effect of age of hardening on strength.

The mine backfill technology requires less strength for the backfill material, and according to China’s Technical Specification For The Total Tailings Paste Backfill (GB/T 39489-2020), the unconfined compressive strength of the backfill material needs to be in the range of 0.2~5 MPa. Figure 5 illustrates the effect of the content of SS and GGBS on the compressive strength of the filled material for curing ages of 7, 14, and 28 days. Figure 5b shows that the compressive strength of the NaOH alkali-activated backfill material increases with the increase in the age of maintenance, and the growth rate of the strength of the backfill material is basically the same from 7 to 28 days, both of which are faster in 7–14 days and slower in 14–28 days. From Figure 5a, it can be seen that the strength increases with increasing GGBS dosage and decreases with increasing SS dosage. The compressive strength reaches a maximum value of 3.78 MPa when GGBS is doped at 16% and SS is doped at 4%, and the compressive strengths all meet the specification requirements. Compared to the reference PG backfill in which the strength is basically around 0.5~2 MPa, this study has greatly improved the strength of PG backfill materials [21,22,42,43]. After being alkali-activated by NaOH, SS and GGBS are highly reactive [44,45], and undergo a series of hydration reactions to produce a large number of C-(A)-S-H gels and ettringite. These hydration products bind the PG not involved in the reaction and fill the structural pores in the filler, thus increasing its compressive strength. As the age of maintenance increases, the reactive substances are gradually consumed, producing progressively more C-(A)-S-H gels, which leads to an increase in strength until the reactive substances are consumed.

CPBM is not mixed with sodium hydroxide, and its strength is only 0.11 MPa at the highest, which is far below the specification requirements (GB/T 39489-2020). SS contains a certain amount of CaO, which can provide an alkaline effect to the slurry and can stimulate the activity of GGBS to a certain extent [46,47,48,49,50]. Since there is still acid remaining in the as-received PG [2,51], the alkaline environment provided by SS is neutralized by the residual acid in the PG, resulting in the failure of the hydration reaction of the slurry. Therefore, the addition of NaOH can well provide an alkaline environment, which is essential for the hydration of SS and GGBS.

### 3.2. XRD Analysis

**Figure 6:**
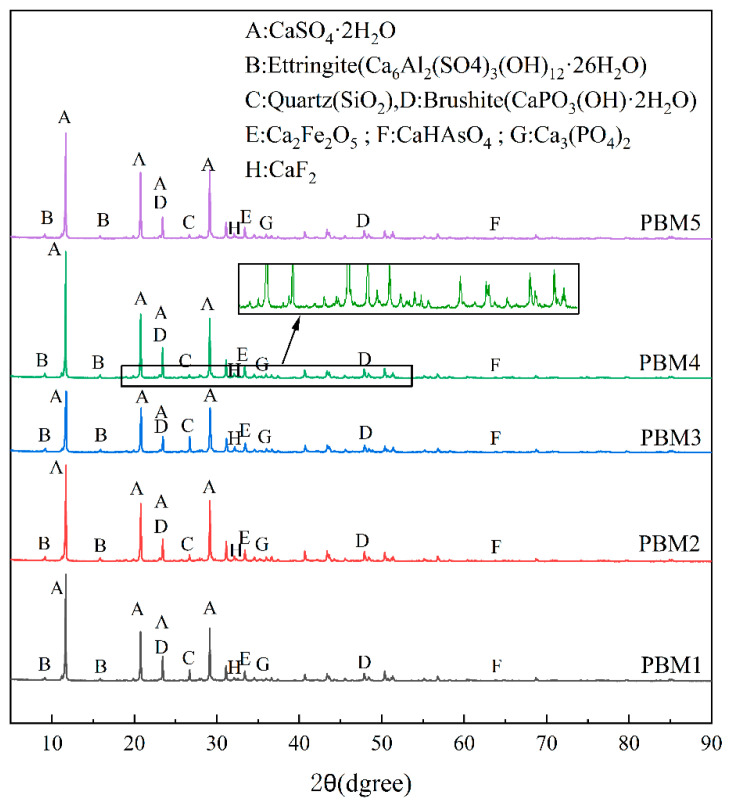
XRD patterns of PBM specimens at different ratios for 28 days.

Figure 6 shows the XRD patterns of PBM specimens at different ratios for 28 days. The diffraction peaks of the XRD patterns of PBM with different ratios are basically the same, indicating that the hydration products in the backfill materials are basically the same, but the intensity of the diffraction peaks corresponding to each crystal is still different. As can be seen in Figure 6, the crystalline minerals in the XRD patterns of the PBM specimens are mainly calcium sulfate dihydrate (CaSO_4_·2H_2_O, PDF#33-0311), ettringite (Ca_6_Al_2_(SO_4_)_3_(OH)_12_·26H_2_O, PDF#41-1451), quartz (SiO_2_, PDF#46-1045), brushite (CaPO_3_(OH)·2H_2_O, PDF#11-0293), srebrodolskite (Ca_2_Fe_2_O_5_, PDF#71-2264), haidingerite (CaHAsO_4_, PDF#74-0141), calcium phosphate (Ca_3_(PO_4_)_2_, PDF#29-0359), and calcium fluoride (CaF_2_, PDF#48-1298). The presence of quartz (SiO_2_) comes from unreacted solid particles. As can be seen from the comparison of Figure 6 with Figure 3, the presence of Ca_2_SiO_4_ was not found in the backfill material, indicating that the Ca_2_SiO_4_ in the SS had a base activation reaction and dissolved. It has been shown [30,52,53,54,55] that SS mainly relies on the reaction of Ca_2_SiO_4_ to provide the gelling capacity. Srebrodolskite detected in the XRD patterns is mainly derived from SS. Ettringite is a hydration product in the gelling system. The early strength of the filled specimens mainly depends on ettringite, which is mainly produced by the reaction of PG with dihydrate of calcium sulfate in PG and Al_2_O_3_ in GGBS involved in the reaction [56]. Haidingerite is a compound produced by the reaction of heavy metal elements with Ca^2+^.

It has been shown that the hump in the XRD patterns in the range of 20°–40° (2θ) is the presence of amorphous gel C-(A)-S-H formation [30,57,58,59,60]. The presence of such humps can be observed in all the XRD patterns of PBM specimens, indicating the generation of amorphous gel substances such as C-(A)-S-H in the filled specimens. C-(A)-S-H gels contribute to the formation of denser microstructures and enhancement of the compressive strength of the filled materials [61,62,63].

### 3.3. SEM-EDS Analysis

**Figure 7:**
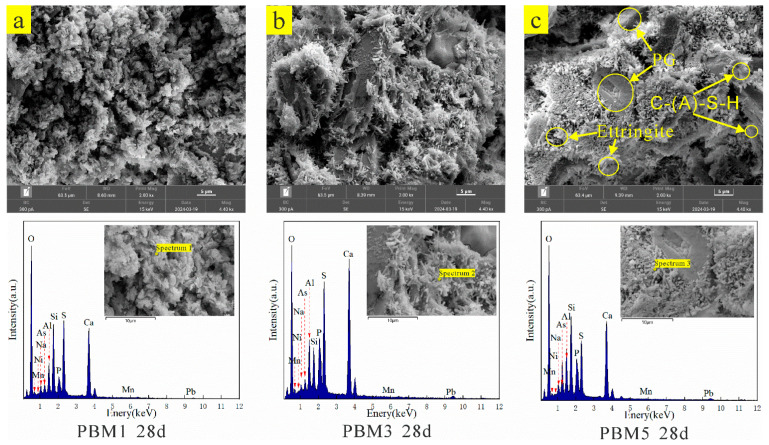
SEM images and EDS results of specimens with different ratios for 28 days. (**a**) PBM1 specimen cured for 28 days, (**b**)PBM3 specimen cured for 28 days, (**c**) PBM5 specimen cured for 28 days.

Figure 7 shows the SEM images of PBM specimens at different ratios of SS to GGBS for 28 days. As in Figure 7a, it can be observed that there are pores, a small amount of C-(A)-S-H gel substance, and ettringite. Its structure is relatively loose, and the pores are larger, less holistic, and produce less gel substance, the bond between particles is weaker, and the macroscopic strength shows lower strength. The lower content of the GGBS hydration reaction produces a less gelling substance, which is insufficient to fill the pores and bond the flake PG. It has been shown that pores can easily cause stress concentrations or shrinkage stress, which are more likely to cause structural damage [64,65]. As shown in Figure 7b, there are pores, amorphous gel material, and ettringite in the PBM3 specimen with a conservation age of 28 days, compared to PBM1, which formed more ettringite and gel material wrapped around unreacted lamellar PG, and the structure is presented to be more compact, but there are more pores, and the macroscopic strength is shown to be larger. As shown in Figure 7c, the microstructure clearly becomes denser and more holistic. This suggests that the increase in GGBS content leads to an increase in activated alumina and silica, which produces more C-(A)-S-H gel material, and as more gel material is formed, it fills in the existing pore structure and binds the residual solid particles together to form a more continuous and dense structure, which reduces porosity and contributes to the increase in strength.

Table 4 shows the 28-day EDS and the corresponding chemical element content occupancy of the PBM specimens at different ratios of SS to GGBS. The EDS test results show that the gel substance mainly contains elements such as O, Al, Si, Ca, and S. The presence of element S indicates the formation of ettringite. The atomic ratios of Ca/Si and Al/Si of the gel substance are (1) 4.23, 2.47, and 1.55; (2) 0.8, 0.73, and 0.69, respectively; in which the Ca/Si ratio gradually decreases with the increase in the content of GGBS; this is because the increase in GGBS brings more silica, which leads to an increase in the content of elemental Si, and makes the Ca/Si in the gel substance atomic ratio increase. The presence of Ca, Al, and Si elemental peaks were all detected in the EDS spectra with high atomic content, indicating C-(A)-S-H gel formation. Na elemental peaks were also detected, indicating that a geopolymerization reaction took place to form N-A-S-H gel [66,67]. The elements As, Ni, Mn, and Pb are mainly derived from PG, and the presence of As, Ni, Mn, and Pb can be seen in the EDS test results, indicating that the heavy metal elements are solidified in the gel product.

### 3.4. Leaching of Toxic Impurities in PBM

#### 3.4.1. Leaching of Element Fluorine in PBM

**Figure 8:**
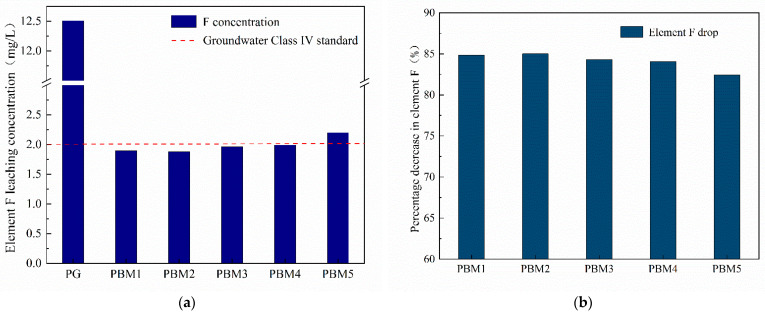
F-element leaching results of the specimen for 7 days: (**a**) Element F leaching concentration; (**b**) Percentage decrease in element F.

Figure 8 shows the results of elemental fluorine leaching from the PBM specimens at a conservation age of 7 days. From the chemical equation for the production of phosphoric acid [2]:

Ca_5_F(PO_4_)_3_ + 5H_2_SO_4_ + 10H_2_O→3H_3_PO_4_ + 5CaSO_4_∙2H_2_O + HF (1)

Fluorine is mainly derived from phosphates (fluorapatite, Ca5(PO4)3F), and most of the fluorine is volatilized to HF gas during phosphoric acid production. However, due to volatilization or incomplete reaction, some of the fluorine remains in the PG and negatively affects the environment, leading to an excess of fluorine in the groundwater of the surrounding environment. The average fluorine-element leaching concentration of phosphogypsum was about 12.5 mg/L, far exceeding the groundwater Class IV standard (2 mg/L). As can be seen in Figure 8a, the concentration of the element fluorine in the leachate decreases substantially when PG is mixed with binders. When the SS content was too low (PBM5), its fluorine leaching concentration was 2.2 mg/L maximum. The leaching concentration of the element fluorine under other ratios is basically below 2 mg/L, which meets the groundwater class IV standard. As can be seen in Figure 8b, the curing efficiency of fluorine-element at different ratios is more than 80%, indicating that utilizing a mixture of GGBS, SS, and PG helps to cure fluorine contamination. It was shown that fluorine ions react with Ca ions in the backfill slurry to form the insoluble compound CaF_2_ [21,22]. As shown in Figure 5, CaF2 was detected in the XRD pattern, which was similar to that of Ref [24]. This further indicates that the F ions were stabilized by reacting to form compounds. In the PBM system, due to the addition of NaOH and SS, with SS containing a certain amount of CaO and therefore providing more Ca^2+^ for the system, NaOH mixing makes the system alkaline, resulting in the increase in Ca^2+^ and OH^−^, which helps the curing of F^−^. The maximum leaching concentration of elemental fluorine in PBM5, which is more than the standard of groundwater class Ⅳ, may be due to the low content of SS and the reduction in the CaO content, which resulted in less Ca^2+^ content in the slurry. Possible chemical equations for the fluorine curing/stabilization process [68]:

Ca^2+^ + 2F^−^ + 2H^+^ + 2OH^−^→CaF_2_ + 2H_2_O (2)

#### 3.4.2. Leaching of Element Phosphorus in PBM

**Figure 9:**
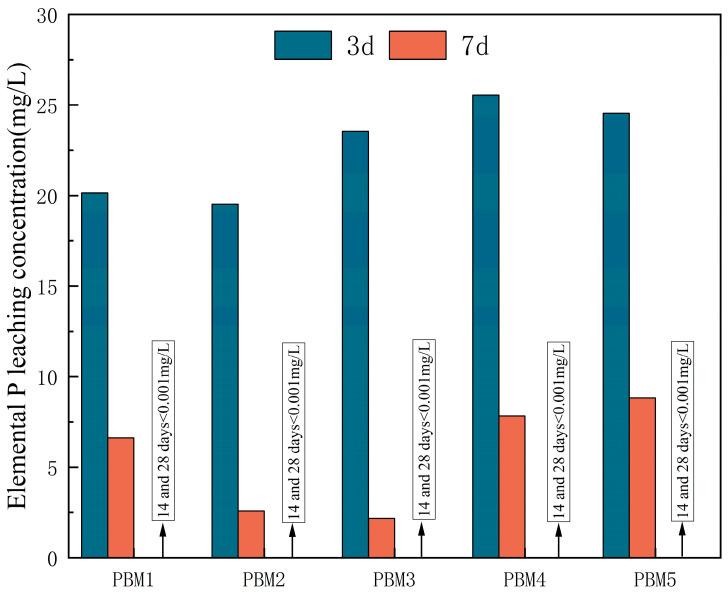
Leaching concentration of elemental P in PBM with age of conservation.

Elemental phosphorus is an important environmental indicator, and Figure 9 shows the leaching concentration of elemental phosphorus for PBM specimens at 3, 7, 14, and 28 days for each ratio. Phosphorus is mainly derived from phosphate, and the leaching concentration of elemental phosphorus in PG exceeds 40 mg/L. From Figure 9, it can be seen that the phosphorus leaching concentration in PBM leachate decreases with the increase in the age of hardening, and the rate of decrease in phosphorus leaching concentration is basically the same in all groups of PBM, all of them have the fastest rate of decrease in 3–7 days, and the rate of decrease slows down in 7–14 days, until the phosphorus in PG is solidified and stabilized. When the age of hardening exceeded 14 days, the phosphorus leaching concentration was detected to be less than 0.001 mg/L, indicating that the PBM system helped to stabilize the phosphorus element.

The main types of phosphate in PG leachate are H_3_PO_4_, H_2_PO^−^, HPO_4_^2−^, and PO_4_^3−^, and the form of phosphate present in the leachate varies according to the pH value [69]. In this study of PBM specimens, due to the addition of NaOH solution as an alkali-activated agent, the main forms of phosphorus in an alkaline environment are PO_4_^3−^ and HPO_4_^2−^ [21,22]. In the PBM system, due to the addition of SS and GGBS, a large amount of Ca^2+^ is provided, and the solidification and stabilization of phosphorus is mainly achieved through the reaction of Ca^2+^ with PO_4_^3−^ and HPO_4_^2−^ to form Ca_2_(PO_4_)_3_ and CaHPO_4_ infusibility substances. As in Figure 5, the presence of Ca_3_(PO_4_)_2_ was examined in the XRD pattern, which was similar to that of Ref. [24], further indicating that the phosphorus was stabilized by reacting to produce the compound form. Possible chemical equations for its curing and stabilization process [68,70]:

3Ca_2+_ + 2PO_4_^3−^→Ca_3_(PO_4_)_2_ (3)

Ca^2+^ + HPO_4_^2−^→CaHPO_4_ (4)

#### 3.4.3. Heavy Metal Leaching

**Figure 10:**
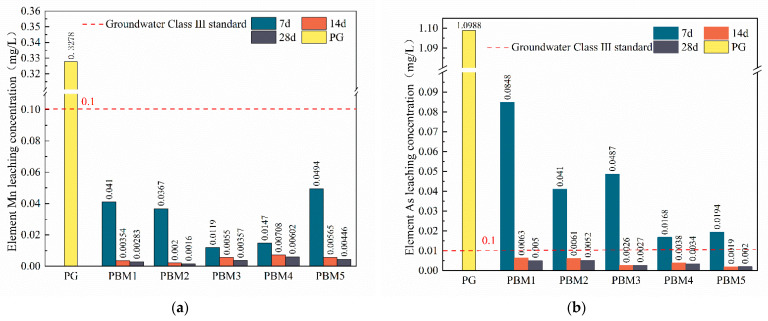

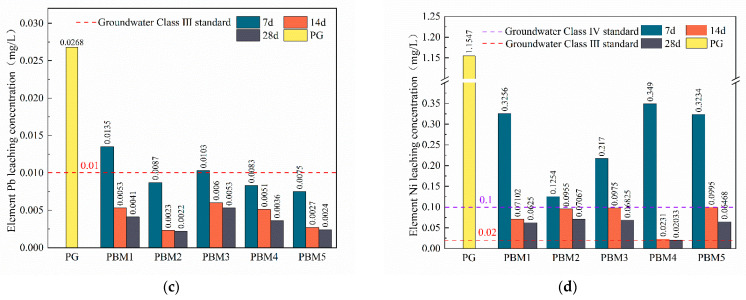
Hazardous element leaching from PBM: (**a**) Mn leaching concentration at different ratios; (**b**) As leaching concentration at different ratios; (**c**) Pb leaching concentration at different ratios; (**d**) Ni leaching concentration at different ratios.

As can be seen from Table 2, the elements in PG with leaching concentrations of heavy metal elements exceeding the groundwater Class III standard are mainly Mn, Pb, As, and Ni. Figure 10 shows the results of heavy metal leaching tests of the specimens at 7, 14, and 28 days, which shows that the heavy metal leaching concentration of the filled specimens with more than 7 days of hardening is significantly reduced and decreases with the increase in the age of hardening. The overall curing effect of the Mn element was the best, and its leaching concentration was much lower than that of the groundwater class III standard (0.1 mg/L) from 7 days, and the maximum Mn leaching concentration was 0.049 mg/L (Figure 10a). Ni has the highest heavy metal content in PG, and it can be seen from Figure 10d that the curing effect of metal elements of Ni at 7 days of maintenance was poor, and the leaching concentration was much higher than that of the groundwater class III standard (0.1 mg/L), and after 14 d of maintenance, Ni leaching concentration significantly decreased, although the leaching concentration was still higher than that of the groundwater class IV standard (0.02 mg/L); after 14 days of maintenance, Ni leaching concentration is obviously reduced, although the leaching concentration is still higher than that of the groundwater III standard, but in line with that of the groundwater IV standard (0.02 mg/L). The leaching concentration of As and some Pb elements of PBM exceeded that of the groundwater class III standard at 7 days of maintenance, and with the increase in maintenance time, the heavy metals were gradually cured, and the leaching concentration decreased, which was lower than that of the class III specification requirements. Through the toxicity leaching test of PBM, the results basically meet the relevant standards, indicating that the whole solid waste is feasible as a curing agent for PG. In previous studies, many of them added cement or quicklime to synergize with other solid wastes as curing agents [20,23,24]. In contrast, the use of all-solid waste in this study reduces the use of cement and quicklime.

From Figure 7, it can be seen that there is a large amount of needle-like Ettringite in PBM, and Ettringite is the main product of the hydration reaction. It has been shown that a large number of Ettringite crystals arranged in a needle-like arrangement can encapsulate heavy metal ions, thus stabilizing heavy metals [70,71,72]. Thus, the decrease in toxic leaching concentration is mainly due to the adsorption and encapsulation of toxic ions by Ettringite, which stabilizes the heavy metal ions. From the analysis of the EDS results of the hydration products in Section 3.3, it is possible to check for toxic elements, proving that the hydration products have a stabilizing effect on these elements. The toxic leaching concentration in Figure 10 decreases with increasing curing time, probably because the specimen produces more gel material in the system with increasing curing time, which facilitates the stabilization of more toxic elements. In addition, heavy metals may be stabilized/solidified by adsorption, and ion exchange, and undergo chemical reactions through hydration products [73,74,75]. C_2_S, C_3_S, and CaO in SS, CaSO_4_ in PG, and Al_2_O_3_, SiO, and CaO in GGBS hydrated to form N-A-S-H, C-(A)-S-H gels and ettringite. The gel product contains a large number of tiny pores that can adsorb heavy metal ions to a large extent. Mn, Pb, As, and Ni ions can be displaced with Al^3+^, Ca^2+^, and SO_4_^2−^ in gel products and ettringite [74,76,77]. Toxic substances may be involved in chemical reactions to generate insoluble compounds and thus achieve stabilization, e.g., HAsO_4_^2+^ can react with Ca^2+^ to CaHAsO_4_. The presence of CaHAsO_4_ compounds was examined in the XRD pattern, as shown in Figure 6.

## 4. Conclusions

In this paper, phosphogypsum-based backfill material (PBM) was prepared using PG, SS, and GGBS. The compressive strength, microstructures, and toxic leaching of PBM were investigated. The heavy metal curing mechanism was discussed and the main conclusions were obtained as follows:

(1) The compressive strength of PBM increased with the increase in GGBS, which increased from 4% to 16%, and the 28 d strength increased from 0.89 MPa to 3.78 MPa, all of which could meet the requirements of backfill technical specification.
(2) The hydration products of PBM are mainly ettringite and C-(A)-S-H gel, and the production of ettringite is conducive to the formation of a network skeleton to enhance the microstructure of PBM, while the C-(A)-S-H gel effectively binds the unreacted PG, and at the same time, fills up the structural pores to form a dense microstructure, which improves the strength.
(3) Toxic leaching showed that fluorine, phosphorus, and heavy metal elements were effectively stabilized and applied in the backfill technology without toxic leaching contamination. The heavy metal curing mechanism is manifested as follows: F^−^ reacts with Ca^2+^ to generate the infusibility compound CaF_2_, phosphorus generates infusibility substances Ca_2_(PO_4_)_3_ and CaHPO_4_ by reacting with Ca^2+^, and the addition of SS can provide the system with more CaO, which is conducive to the stabilization of fluorine and phosphorus. Heavy metals can be chemically adsorbed or ionically displaced by ettringite and gel products to achieve the curing purpose.
(4) The use of alkali-activated all-solid waste as a curing agent for PG backfill material was shown to provide a solution for recycling large quantities of industrial solid waste into utilization to alleviate the pressure of environmental pollution based on the mechanical properties and environmental suitability of PBM.
(5) However, 20% of the binder was used in this study compared to about 10% used for common backfill materials, which may be attributed to the high acid and sulfate content of PG. The leaching concentrations of F ions and some pollutant elements were higher than the groundwater class III standard. Therefore, further studies are needed to develop effective curing contaminants and low-cost binders for PG-based CPB.

## Figures and Tables

**Table 1:** Chemical composition of materials.

Materials	Chemical Composition						
	SiO_2_	CaO	Al_2_O_3_	Fe_2_O_3_	MgO	SO_3_	Others
PG	2.45	36.59	0.34	0.34	0	59.78	0.5
GGBS	34.5	34	17.7	1.03	6.01	1.64	5.58
SS	16.22	42.19	6.33	18.93	8.37	0.97	6.99

**Table 2:** Hazardous element content of PG.

Hazardous Element Content (mg/L)	Mn	Pb	Se	As	Ni	Be	Cu	Zn	Cd	Ba
PG-1	0.5442	0.0281	0.0058	1.1537	0.8124	0.0004	0.0441	0.1365	0.0010	0.0105
PG-2	0.1147	0.0257	0.0013	1.5548	1.1085	0.0002	0.0203	0.1748	0.0002	0.0076
PG-3	0.3245	0.0265	0.0094	0.5878	1.5432	0.0006	0.0616	0.0787	0.0009	0.0119
Average concentration	0.3278	0.0268	0.0055	1.0988	1.1547	0.0004	0.0420	0.1300	0.0007	0.0100
Class III Standard	0.1000	0.0100	0.0100	0.0100	0.0200	0.0020	1.0000	1.0000	0.0050	0.7000

**Table 3:** Experiment scheme.

Sample ID	Raw Material (%)			Molar Concentration of Alkali-Activated (mol/L)	Liquid–Solid Ratio	Age of Hardening (Days)
	PG	SS	GGBS			
PBM1	80	16	4	1	0.4	7, 14, 28
PBM2	80	13	7	1	0.4	
PBM3	80	10	10	1	0.4	
PBM4	80	7	13	1	0.4	
PBM5	80	4	16	1	0.4	
CPBM	80	10	10	0	0.4	

**Table 4:** Percentage of atoms obtained by EDS (%).

Elemental		O	Na	Al	Si	P	S	Ca	Mn	Ni	As	Total
Spectrum 1	Wt%	54.18	1.27	3.27	8.08	1.12	11.49	18.46	0.14	0.03	0.87	100
	At%	71.69	1.17	2.56	6.08	0.77	7.57	9.73	0.06	0.01	0.24	100
Spectrum 2	Wt%	47.75	0.79	4.24	3.54	1.21	12.09	26.19	0.19	0.22	0.87	100
	At%	67.82	0.78	3.56	2.86	0.88	8.54	14.8	0.08	0.1	0.26	100
Spectrum 3	Wt%	50.89	1.72	4.64	8.97	1.06	8.17	19.02	0.17	0.18	2.36	100
	At%	69.78	1.63	3.76	6.99	0.75	5.58	10.38	0.07	0.07	0.69	100

## Data Availability

Data are contained within the article; further inquiries can be directed to the corresponding authors.
